# Targeted nutritional intervention with enhanced recovery after surgery for carotid endarterectomy: A prospective clinical trial

**DOI:** 10.3389/fnut.2023.951174

**Published:** 2023-04-13

**Authors:** Yu-Qian Li, Xiao-Peng Qu, Li-Wei Peng, Jie-Yuan An, Xin-Wei Liu, Yue Zhang, Chao Wang, Xue Jiang, Li Gao, Gang Li, Da-Li Wang, De-Chang Zhao, Yan Qu, Bei Liu

**Affiliations:** ^1^Department of Neurosurgery, Tangdu Hospital, Airforce Military Medical University, Xi'an, China; ^2^The Third Brigade, Basic Medical Science Academy, Airforce Military Medical University, Xi'an, China

**Keywords:** carotid endarterectomy, enhanced recovery after surgery, nutrition, rehabilitation, ischemic stroke

## Abstract

Ischemic stroke is the most common cerebrovascular disease, and vascular obstruction is an important cause of this disease. As the main method for the management of carotid artery stenosis, carotid endarterectomy (CEA) is an effective and preventive treatment measure in ischemic cerebrovascular disease. This study aims to propose the application of a new enhanced recovery after surgery (ERAS) nutritional support regimen in CEA, which can significantly improve the perioperative nutritional status of patients. A total of 74 patients who underwent CEA were included and randomly divided into two groups: 39 patients received nutritional therapy with the ERAS protocol (ERAS group) and 35 patients received routine perioperative nutritional support (control group). Our results showed that the levels of major clinical and biochemical parameters (albumin, hemoglobin, creatinine, calcium and magnesium levels, etc.) in the ERAS group were significantly higher than those in the control group after surgery (*p* < 0.05). Additionally, patients in the ERAS group had dramatically shorter postoperative length of stay and reflected higher mean satisfaction at discharge (*p* < 0.001). Moreover, no statistically significant differences were observed in postoperative complication rates and Mini-mental State Examination scores at discharge. The emergence of this neurosurgical ERAS nutritional support program can effectively intervene in perioperative nutritional status, and notably reduce postoperative hospital stays.

## Introduction

The causes of ischemic stroke are extremely complex and diverse, and mainly result from thromboembolic occlusion of the major cerebral artery or its branches and severe narrowing (stenosis) of the carotid artery (1). The two major approaches, neuroprotection and improvement of cerebral blood circulation, are used to treat ischemic stroke, the latter of which uses thrombolytic drugs or mechanical devices to recanalize occluded vessels (2, 3). Carotid endarterectomy (CEA) is a surgical procedure for patients with vascular stenosis or obstruction that can remove the carotid intima thickened atherosclerotic plaque to prevent stroke caused by plaque peeling off (4–6). Carotid surgery treatment may reduce the risk of stroke and the incidence of cerebral infarction, and effectively control postoperative and long-term stroke recurrence and mortality (7). However, patients undergoing CEA surgery are often at an advanced age and have comorbid organ diseases, along with degeneration of body functions and other factors. Therefore, postoperative weakness, metabolic disturbance and malnutrition are prone to occur after invasive surgery under general anesthesia due to the stress response of surgical trauma and postoperative fasting. Based on this situation, our research group put forward the application of a nutritional protocol for enhanced recovery after surgery (ERAS) in CEA.

ERAS refers to the implementation of various effective methods in the perioperative period to reduce the complications of patients undergoing surgery and speed up the recovery of patients (8, 9). ERAS pays attention to reducing the perioperative stress response of surgical patients, including physical and psychological stress. Although there is no report on the application of ERAS in CEA for nutritional support, referring to the clinical application of other disciplines, it is expected to potentially improve the nutritional status of patients during the perioperative period, enhance the immune function of patients, and promote postoperative recovery (10–13).

To the best of our knowledge, the ERAS protocol for CEA, especially with regard to its nutrition domain, has not been established. Recently, our research group developed a multidisciplinary ERAS protocol for CEA based on the best available evidence. The aim of the present study was to prospectively propose a novel, multidisciplinary, evidence-based, neurosurgical ERAS nutritional protocol for CEA. We wished to evaluate the safety and effectiveness of the ERAS nutritional regimen, and to expectantly evaluate there was a significant postoperative improvement in physical condition and recovery in patients compared with those receiving standard care in our institution.

## Materials and methods

### Patient recruitment

This study was carried on between February 2020 and July 2021 at the Department of Neurosurgery, Tangdu Hospital, Air Force Medical University (Xi’an, China) and was registered with the Chinese Clinical Trial Registry (ChiCTR2000029570). The randomized control trial was approved by the Institutional Human Research and Ethics Committee of Tangdu Hospital. All patients were provided with all the information concerning the study, including detailed explanations and written notifications. Informed and signed consent was obtained from all patients to participate and all patients with CEA were screened for eligibility. Patients that required emergency surgery, had serious consciousness and movement disorders before surgery, required emergency surgery pathologically, or who had other confounding factors that may affect postoperative recovery (such as paralysis, spinal deformity, autoimmune diseases, myocardial infarction, serious infection, liver or kidney dysfunction or serious psychological or mental diseases) were excluded.

A total of 74 patients who met the selection criteria were randomized into either an ERAS group or control group. The selection sequence was computer generated and the results were reviewed by a statistician, which ensured the objectivity and randomness of the experiment. First, 35 patients were placed in the control group and received routine perioperative care according to the practice mode of the institution. The remaining 39 patients were assigned to the ERAS group and received treatment according to new ERAS nutritional protocol described in this study. The researchers responsible for the follow-up visit and the surgeons were all masked to treatment assignment during the study phase of CEA. Through these measures, the study was not affected by subjective human factors.

### Nutritional risk screening

The screening tool we used was nutritional risk screening 2002 (NRS 2002), which was proposed by European Society for Parenteral and Enteral Nutrition (ESPEN) guidelines on the basis of analysis of controlled clinical trials (14–16). A total score greater than 3 indicated that the patient was malnourished or at risk of nutrition and should avail of nutritional support. If the score was 0–3, patients only had a slight risk were excluded from the study. Their status was reviewed weekly and these patients were received no nutritional support.

### Nutritional protocol for ERAS and conventional care

The nutrition plan for this ERAS was designed for patients undergoing CEA, based on concepts from other established plans and drawing on extensive and current evidence-based support for perioperative nutritional interventions. We have studied traditional nutritional support programs and improved them according to the specific conditions of individual patient, and creatively proposed a new set of ERAS nutritional programs. Briefly, our protocol includes the following aspects: (1) preoperative management and assessment, which aimed at correcting malnutrition, improving nutritional status and optimizing body composition. A nutritional support working group was established to cooperate closely with clinicians and support staff from the ultrasound, anesthesiology, inpatient and surgical care, and nutritional services departments. Patients in the ERAS group abstained from solid food for 6 h before surgery, took no more than 400 ml of carbohydrates orally 2 h before surgery and had their fasting blood glucose and preoperative blood glucose monitored. Meanwhile, for malnourished patients, preoperative nutritional support was required. The preferred method was enteral nutrition (EN) support 7–10 days before surgery, usually with oral nutrition supplementation. However, if the patient had an intake disorder, tube feeding was required. (2) Intraoperative surgery and anesthesiology management, although not the key to the ERAS nutritional program, was a standard intraoperative measure and an important prerequisite for ERAS. Firstly, selected the appropriate surgical position and approach, and designed a reasonable surgical incision. Secondly, ropivacaine was given before surgery for subcutaneous local anesthesia (when the operation time > 3 h, anesthesia was given again), concomitantly, general anesthesia combined with regional nerve block anesthesia was selected, and the systemic application of opioids was reduced. Next, it was necessary to strictly control arterial blood pressure and maintain the stability of cerebral flow during the operation, and monitor end-tidal carbon dioxide to prevent hyperventilation. Finally, optimized the suture of the incision and avoided routine drain placement. (3) Postoperative nutritional support: patients in the ERAS group were given parenteral nutrition (PN) and EN support according to their own gastrointestinal conditions. The timing and dose of early postoperative eating or EN were determined according to the patient’s gastrointestinal function and tolerance. Liquid food was taken 6 h after the operation, after which the patients could be changed to semi-liquid food after the intestinal ventilation was restored, and the intake could then be gradually increased according to the tolerance of the gastrointestinal tract. During this period, the oral administration or tube feeding of EN solution, as the core of ERAS nutritional care, played an irreplaceable role in postoperative nutritional support. In particular, when the patient was not suitable to receive EN, nutrients were provided by continuous infusion through the peripheral intravenous route of PN. In cases where EN could not be initiated, PN was supplemented as soon as possible if the patient was at high nutritional risk (NRS score ≥ 5). Supplemental PN was administered if EN intake and protein levels remained below 60% of target after 7–10 days of EN treatment. In addition, blood glucose continued to be strictly monitored postoperatively to keep it within the ideal range of 7.77–9.99 mmol/L.

**Figure 1:**
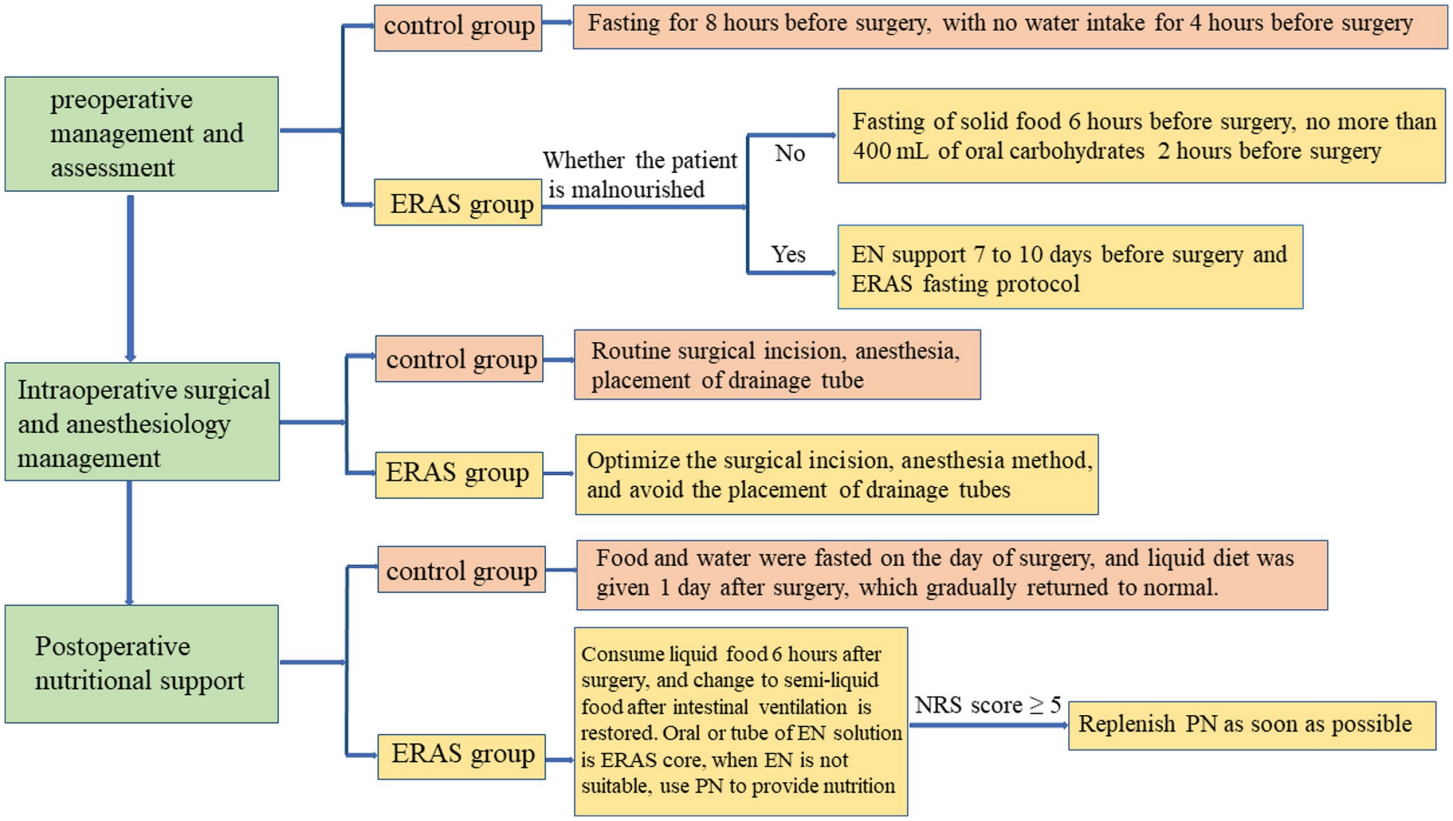
Summary of nutritional interventions in the two groups.

Correspondingly, the control group adopted the traditional regimen, fasting for 8 h before surgery, with no water intake for 4 h before surgery. A liquid diet was given 1 day after surgery, which gradually returned to normal. It should be noted that the measures presented above are only one part of the ERAS scheme and must be highly compatible with other steps of ERAS in order to achieve better clinical treatment effect (Figure 1).

Patients received conventional perioperative care in our unit. Preoperative care mainly included psychological care of patients, ward environmental protection, advice on smoking and alcohol cessation, application of preventive antibiotics and antithrombotic therapy, etc. Meanwhile, postoperative care, including monitoring of vital signs every 1–2 h, nebulization of the airway, sleep management and setting discharge criteria was also implemented. Patients were also advised to perform appropriate rehabilitation exercise, which was conducive to the patient’s positive mood and a more ideal prognosis.

### Health guidance after discharge

After discharge, it was recommended that patients limit heavy physical activity and avoid strenuous exercise for 3 to 4 weeks. Also, patients were instructed to maintain emotional stability and avoid excessive tension, excitement, or mood swings. In addition, it was also essential to develop good living habits, such as quitting smoking and drinking, eating a reasonable diet, and resting frequently. In terms of nutrition, for most surgical patients, low-salt, low-fat and easily digestible food was recommended to maintain a balanced diet and satisfy the body’s needs for various nutrients. If the patient lose weight significantly after surgery, it was recommended to increase the intake of calories and protein to meet the needs of rehabilitation. In particular, oral nutritional supplements were an important component of post-discharge dietary plans for surgical patients. For severely malnourished patients and patients with long postoperative hospital stay or intensive care units stay, oral nutritional supplements were instructed to be used for 3 to 6 months after surgery. Nursing personnel instructed the patient to self-observe bleeding tendency and take medication as prescribed. Finally, the patients needed to actively cooperate with telephone follow-up and outpatient follow-up.

### Data collection and observation indicators

Preoperatively, demographic variables including age, sex, height, weight, body mass index, education level, occupational status, marital status, American Society of Anesthesiologists grade, and patient comorbidities (smoking, diabetes, hypertension, hypercholesterolemia, etc.) were recorded clinically. Biochemical and clinical parameters, including albumin, hemoglobin, liver and kidney function, and electrolytes, were also measured by preoperative venous blood collection. During the operation, blood glucose, blood pressure, pulse, oxygen saturation, central venous pressure, body temperature, end-tidal carbon dioxide, and respiratory rate were monitored. Postoperatively, peripheral fasting venous blood was extracted on the first and third postoperative days to determine biochemical and clinical parameters. Furthermore, the patients’ bowel movements were observed and recorded, as were other conditions during the nutritional support treatment. Clinical outcome variables comprised readmission, reoperation, postoperative surgical and non-surgical complications, as well as functional recovery [Karnofsky performance status (KPS)] at discharge and at 30-days follow-up. The primary endpoint was the postoperative length of stay (LOS), and the secondary endpoints included postoperative complications, postoperative quality-of-life (QoL), medical cost, readmission, and evaluation of patient satisfaction. At the same time, the symptoms of each group were observed during the treatment, and the prognosis was determined at the time of discharge (17, 18).

### Statistical analysis

The data analysis was performed using the SPSS (Ver. 19, IBM Corp., Armonk, NY, United States). Descriptive statistics were used to define baseline characteristics. The Kolmogorov–Smirnov test was used to identify the normal distribution of the variables. Group differences with continuous data with normal distribution were statistically examined using the Student’s *t*-test, while data without normal distribution were analyzed using the Mann–Whitney *U*-test. Readmission, complications, and mortality were analyzed using the chi-square test (with/without Yates correction) or Fisher’s accurate test. The sample size was powered to be 58 patients in each group based on the hypothesis that the primary outcome (postoperative LOS) would be reduced by 25% with a power of 80% and significance of 5%. Assuming a maximal dropout rate of 20%, the final sample size was determined as 74 patients per arm. In turn, analysis was planned when the minimal number of the predefined sample size was met. The data were considered to be statistically significant if *p* < 0.05.

## Results

### Baseline characteristics

**Figure 2:**
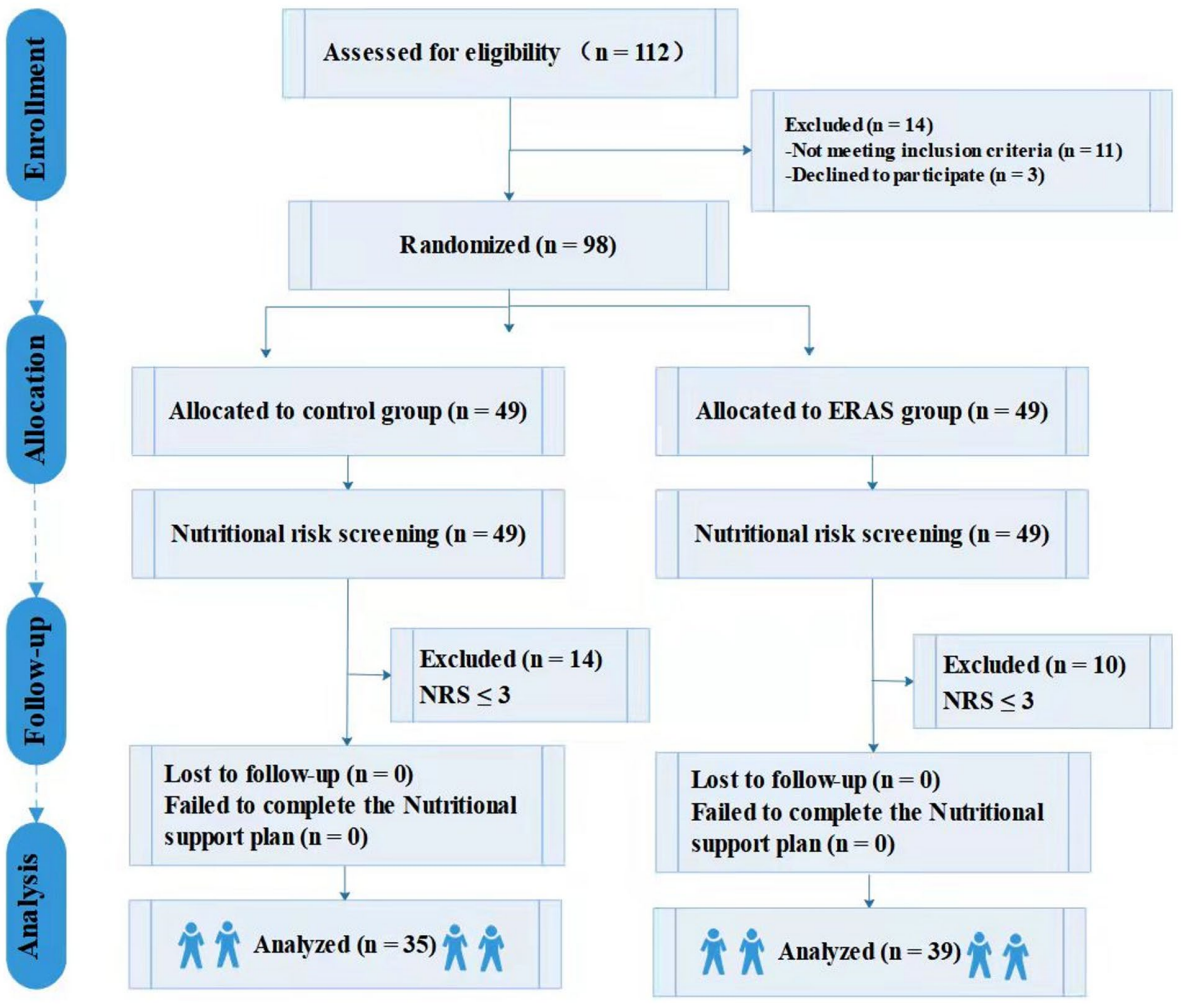
Flow diagram for study participants.

Between February 2020 and July 2021, a total of 112 patients from our hospital were enrolled in the present study. After exclusion, a total of 74 patients (35 in the control group and 39 in the ERAS group) were included in the analysis (Figure 2). The demographic and clinical characteristics (including sex, mean age, mean BMI, ASA grade, and marital status, etc.) of the two groups of patients were not significantly different (*p* > 0.05). Concomitant diseases such as cerebral infarction, sequelae of cerebral infarction, cardiac/hypertension, diabetes mellitus, hypercholesterolemia, liver/gallbladder, lung, and miscellaneous were equally distributed between the two groups. In terms of nutrition, most patients both groups had normal nutritional status. Both groups of patients underwent CEA by the same experienced surgical team, and all patients received the assigned intervention. Characteristics of patients are shown in Table 1.

**Table 1:** Sociodemographic and clinical features [Mean + SD, *n*(%)].

Variable	Control group (*n* = 35)	ERAS group (*n* = 39)	*p*-value
Mean age (year)	72.09 ± 10.05	70.41 ± 8.42	0.438
Gender (n)			0.116
Male	21 (60.00%)	30 (76.92%)	
Female	14 (40.00%)	9 (23.08%)	
Mean BMI(kg/m^2^)	20.90 ± 1.02	21.36 ± 1.94	0.163
Education (*n*)			0.417
No education/primary school	8 (22.86%)	11 (28.21%)	
Secondary school/high school	13 (37.14%)	9 (23.08%)	
College/more than college	14 (40.00%)	19 (48.72%)	
Occupation (*n*)			0.84
Employed	9 (25.71%)	10 (25.64%)	
Unemployed	11 (31.43%)	9 (23.08%)	
Retired	9 (25.71%)	11 (28.21%)	
Home maker	6 (17.14%)	9 (23.08%)	
Marital status (*n*)			0.805
Unmarried (single/divorced)	3 (8.57%)	4 (10.26%)	
Married	32 (91.43%)	35 (89.74%)	
ASA grade (*n*)			0.81
ASA I	9 (25.71%)	11 (28.21%)	
ASA II	26 (74.29%)	28 (71.79%)	
Concomitant diseases (*n*)			
Cerebral infarction	8 (22.86%)	9 (23.08%)	0.982
Sequelae of cerebral infarction	2 (5.71%)	3 (7.69%)	0.735
Cardiac/hypertension	25 (71.43%)	30 (76.92%)	0.589
Diabetes mellitus	23 (65.71%)	27 (69.23%)	0.747
Smoker	4 (11.43%)	9 (23.08%)	0.189
Hypercholesterolemia	27 (77.14%)	32 (82.05%)	0.6
Liver/gallbladder	3 (8.57%)	5 (12.82%)	0.714
Lung	3 (8.57%)	2 (5.13%)	0.662
Miscellaneous	4 (11.43%)	3 (7.69%)	0.701
Nutrition (*n*)			0.874
Normal	31 (88.57%)	35 (89.74%)	
Mild malnutrition	1 (2.86%)	2 (5.13%)	
Moderate malnutrition	2 (5.71%)	1 (2.56%)	
Severe malnutrition	1 (2.86%)	1 (2.56%)	

ASA, American Society of Anesthesiologists grade.

### Surgery characteristics

The main surgical results were shown in Table 2. The differences in mean duration of surgery, cross-clamping time, carotid plaque size, blood transfusion, lateral location, and blood loss >300 ml between the two groups were not remarkable. The mean duration of surgery of the ERAS group was 150.08 ± 19.89 min and that of the control group was 149.11 ± 21.71 min (*p* = 0.843). For intraoperative monitoring of CEA patients, blood loss is an important indicator. There were 9 and 11 patients whose blood loss volume was over 300 ml in the ERAS group and control group, respectively (*p* = 0.419). Additionally, two patients in the ERAS group required blood transfusion (i.e., red blood cell and blood plasma transfusions) during the operation, while none of the patients in the control group required a transfusion.

**Table 2:** Surgery characteristics [Mean + SD, *n*(%)].

Variable	Control group (*n* = 35)	ERAS group (*n* = 39)	*P*-value
Lateral location (*n*)			0.451
Right	21 (60.00%)	20 (51.28%)	
Left	14 (40.00%)	19 (48.72%)	
Mean duration of surgery (min)	149.11 ± 21.71	150.08 ± 19.89	0.843
Cross-clamping time (min)	21.29 ± 3.97	19.67 ± 5.63	0.162
Carotid plaque size (cm)	2.67 ± 0.57	2.79 ± 0.52	0.328
Blood loss > 300 mL (*n*)	11 (31.43%)	9 (23.08%)	0.419
Blood transfusion (*n*)	0 (0.00%)	2 (5.13%)	0.174

### Clinical and biochemical parameters

Table 3 mainly described the changes in the content of various substances in the blood before and after surgery. There was no significant difference in the levels of various nutrients between the two groups before surgery. However, at postoperative day (POD) 1, there was a significant trend toward an increase of albumin, hemoglobin and calcium in the ERAS group compared with the control group (*p* < 0.001), which continued at POD 3 (*p* < 0.05). The overall pattern of creatinine and magnesium levels was similar in ERAS group, which increased at POD 1 and then decreased over the next couple of days. In the control group, creatine and magnesium was increased at POD 1 and decreased to the preoperative level at POD 3, while the contents of other substances were decreased at POD 1 and increased at POD 3.

**Table 3:** Laboratory characteristics [Mean + SD].

Variable	Control group (n = 35)	ERAS group (n = 39)	*P* value
**Albumin (mg)**			
Pre-operation	37.78 ± 2.63	37.10 ± 2.76	0.286
POD 1	37.23 ± 2.81	39.73 ± 3.00***	<0.001
POD 3	40.09 ± 2.46	41.93 ± 2.44*	0.002
**Hemoglobin (g/L)**			
Pre-operation	122.60 ± 8.01	124.29 ± 7.44	0.351
POD 1	121.71 ± 7.38	130.83 ± 4.79***	<0.001
POD 3	132.20 ± 6.28	135.59 ± 6.93*	0.032
**Cholesterol (mmol/L)**			
Pre-operation	6.30 ± 1.85	6.32 ± 1.64	0.959
POD 1	6.14 ± 1.81	6.54 ± 1.58	0.322
POD 3	6.54 ± 1.74	6.45 ± 1.49	0.809
**Blood urea nitrogen (mmol/L)**			
Pre-operation	5.77 ± 1.31	5.40 ± 1.23	0.207
POD 1	5.55 ± 1.33	5.75 ± 1.44	0.543
POD 3	6.05 ± 1.43	5.97 ± 1.27	0.794
**Creatinine (umol/L)**			
Pre-operation	74.91 ± 12.89	77.60 ± 11.76	0.35
POD 1	78.47 ± 12.62	79.71 ± 11.48	0.66
POD 3	75.51 ± 13.44	78.91 ± 12.10	0.256
**Calcium (mmol/L)**			
Pre-operation	2.37 ± 0.05	2.35 ± 0.08	0.185
POD 1	2.31 ± 0.05	2.45 ± 0.09***	<0.001
POD 3	2.45 ± 0.13	2.53 ± 0.08*	0.003
**Magnesium (mmol/L)**			
Pre-operation	0.89 ± 0.10	0.88 ± 0.09	0.517
POD 1	0.91 ± 0.08	0.92 ± 0.10	0.818
POD 3	0.88 ± 0.09	0.88 ± 0.10	0.82
**Phosphorus (mmol/L)**			
Pre-operation	1.16 ± 0.10	1.16 ± 0.10	0.747
POD 1	1.15 ± 0.12	1.19 ± 0.10	0.211
POD 3	1.16 ± 0.10	1.16 ± 0.09	0.99

POD, Postoperative day.

^*^P < 0.05; ^***^P < 0.001.

### Postoperative hospital stays and hospitalization expenses

Evaluation of LOS, cost, and postoperative recovery were shown in Table 4. The data showed that LOS was significantly lower in the ERAS group than in the control group (4.31 ± 0.98 days vs. 6.71 ± 2.09 days, *p* < 0.001). Additionally, there was also a significant difference in overall cost between the two groups (2.43 ± 0.18 10,000 yuan in the ERAS group vs. 2.57 ± 0.26 10,000 yuan in the control group, *p* < 0.05).

**Table 4:** Postoperative recovery [Mean + SD].

Variable	Control group (*n* = 35)	ERAS group (*n* = 39)	*P-*value
Postoperative LOS(day)	6.71 ± 2.09	4.31 ± 0.98***	<0.001
Overall cost, 10,000 (Yuan; Chinese Yuan Renminbi)	2.57 ± 0.26	2.43 ± 0.18*	0.017
Overall satisfaction(score)	80.31 ± 4.04	89.82 ± 3.52***	<0.001
Information	15.80 ± 2.61	17.44 ± 1.68***	0.002
Medical care	17.23 ± 1.82	18.46 ± 1.23***	<0.001
Nursing care	17.49 ± 1.80	18.95 ± 1.07***	<0.001
Enhanced recovery	13.49 ± 2.85	18.26 ± 1.48***	<0.001
Comfort and others	16.31 ± 2.41	16.72 ± 2.14	0.448
**QoL (score)**			
Pre-operation	70.29 ± 7.59	70.15 ± 7.21	0.939
Discharge	67.71 ± 5.20	73.00 ± 3.93***	<0.001
POM 3	77.60 ± 8.37	80.74 ± 7.52	0.093
**MMSE (score)**			
Pre-operation	18.60 ± 4.95	19.38 ± 5.28	0.513
Discharge	20.54 ± 4.53	22.97 ± 4.40*	0.022
POM 3	24.83 ± 3.61	25.44 ± 3.80	0.484
GSI POM 3(score)	4.54 ± 2.31	5.95 ± 2.08*	0.007
KPS(score)	90.00 ± 3.83	89.95 ± 3.41	0.952

KPS, Postoperative Karnofsky performance status; Postoperative LOS, length of stay, days; QoL, quality of life; POM, Postoperative month; MMSE, Mini-mental State Examination; GSI POM 3, grip strength improvement in 3 months after surgery. ^*^P < 0.05; ^***^P < 0.001.

### Assessment of patient satisfaction

**Figure 3:**
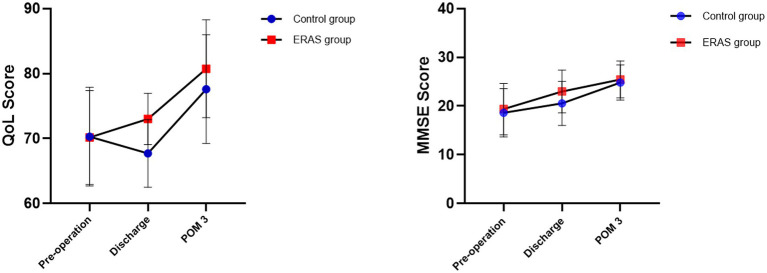
Comparison of MMSE and QoL scores between the two groups.

All patients completed the discharge satisfaction survey questionnaire. The figure below showed the changes and differences in QoL and Mini-mental State Examination (MMSE) scores between the two groups before surgery, at discharge, and at postoperative month (POM) 3 (Figure 3). The mean overall satisfaction of patients in the ERAS group at discharge was significantly higher than that of the control group (89.82 ± 3.52 score vs. 80.31 ± 4.04 score). Similarly, there were differences in satisfaction between the two groups, with obvious differences in medical care (18.46 ± 1.23 score in the ERAS group vs. 17.23 ± 1.82 score in the control group), nursing care (18.95 ± 1.07 score in the ERAS group vs. 17.49 ± 1.80 score in the control group), and enhanced recovery (18.26 ± 1.48 score in the ERAS group vs. 13.49 ± 2.85 score in the control group; *p* < 0.001).

There was little difference in QoL and MMSE scores between the two groups in preoperative period (*p* > 0.05), but differences were evident after discharge. At discharge, the QoL score of the ERAS group was 73.00 ± 3.93 score and that of the control group was 67.71 ± 5.20 score (*p* < 0.001). However, at POM 3, the QoL and MMSE scores of two groups showed no statistical difference (*p* > 0.05; Figure 3).

Follow-up results showed that survey satisfaction in the ERAS group was significantly higher than that in the control group (*p* < 0.001). There were significant differences in grip strength improvement at POM3 between the two groups (5.95 ± 2.08 score in the ERAS group vs. 4.54 ± 2.31 score in the control group, *p* = 0.007). However, no remarkable difference between the two groups was noted in the other outcomes relating to the KPS score (90.00 ± 3.83 score in the ERAS group vs. 89.95 ± 3.41 score in the control group, *p* = 0.952). Detailed patient satisfaction scores according to each module are shown in Table 4.

### Postoperative complications after discharge

Table 5 showed the postoperative complications. Stroke, as an important complication after CEA, occurred in one patient (2.56%) in the ERAS group and two patients (5.71%) in the control group (*p* = 0.493). Two patients in the ERAS group and none in the control group had deep vein thrombosis (*p* = 0.130). None of the patients experienced symptoms of dyspnea or surgical site infection/subcutaneous effusion in the ERAS group. Additionally, postoperative nausea and vomiting intensity scale and nausea visual analog scale were performed postoperatively in both groups, but the proportions of patients with mild, moderate, and severe disease varied.

**Table 5:** Postoperative complications *n* (%).

Variable	Control group (*n* = 35)	ERAS group (*n* = 39)	*P*-value
Mortality (*n*)	0	0	—
PONV VAS (*n*)			0.101
Mild (0–4)	22 (62.86%)	33 (84.62%)	
Moderate (5–6)	9 (25.71%)	4 (10.26%)	
Severe (7–10)	4 (11.43%)	2 (5.13%)	
Dyspnea (*n*)	0(0.00%)	0 (0.00%)	—
Surgical site infection/subcutaneous effusion (*n*)	1 (2.86%)	0 (0.00%)	0.288
Stroke (*n*)	2 (5.71%)	1 (2.56%)	0.493
Cardiovascular (*n*)	0 (0.00%)	1 (2.56%)	0.340
Gastrointestinal (*n*)	1 (2.86%)	1 (2.56%)	0.938
Urinary tract (*n*)	2 (5.71%)	1 (2.56%)	0.493
DVT (*n*)	0 (0.00%)	2 (5.71%)	0.130

PONV, postoperative nausea and vomiting; VAS, Visual Analog Scale; DVT, deep vein thrombosis.

## Discussion

The traditional nutritional regimen includes fasting before surgery, a liquid diet on the first postoperative day, after which the patient gradually transition to a normal diet. Preoperative fasting depletes the body’s carbohydrate reserves, resulting in reduced preoperative comfort. In addition, fasting may change the body’s endocrine and metabolic response and reduce the body’s ability to resist stress after surgery, thereby increasing postoperative complications. Thus, it can be seen that conventional nutritional support for the patient remains problematic, unsystematic and not fully aligned with clinical care and other perioperative steps.

Due to the importance of the perioperative nutritional status of CEA patients, nutritional improvement measures for patients have become diverse and complex (19, 20). It is of great significance to propose a new and more reasonable perioperative nutritional therapy based on existing mature experiences of medical staff, research literature, and research progress. Briefly, the nutritional measures combined both EN and PN, with EN as the key factor to improve the measures and the main nutritional mode. Compared with the control group, which adopted a conventional nutritional regimen, the ERAS group had better preoperative mental states, visual field vision, language, body activity, and limb muscle strength. It is beneficial to the success of the operation, and significantly promotes the postoperative intervention and nursing, as well as the rehabilitation of patients.

The results of this study showed that serum albumin decreased in patients undergoing CEA surgery within a short period of admission. After 3 days of nutritional support treatment, serum albumin and hemoglobin increased more significantly in the ERAS group than in the control group, indicating that the ERAS group was more conducive to protein synthesis. In terms of specific postoperative physiological parameters, such as blood albumin, hemoglobin, cholesterol, and creatinine, among others, the ERAS group had better results than those of the control group, which may reflect the result’s wider significance that patients in the ERAS group had increased immunity, better body function, reduced complications, and shorter length of hospital stay. According to the personalized evaluation of patients, the satisfaction of the ERAS group was also much higher than that of the control group. Indeed, the patients in the ERAS group had better clinical compliance of postoperative follow-up. Thus, the nutritional measures had a good effect on the patients’ physical condition and significantly improved postoperative recovery. Furthermore, it had a positive effect on patients’ subjective feelings and inner emotions.

In recent years, it has been recognized that the gastrointestinal tract is not only an organ of digestion and absorption, but an important immune organ (21, 22). Based on this, the advantages of EN are not only reflected in the direct absorption and utilization of nutrients through the intestine, more physiological, convenient administration and low cost, but helped to maintain the integrity of intestinal mucosal structure and barrier function (23, 24). The ESPEN guidelines propose that normal food intake or EN should start early after surgery (25). An analysis was conducted to investigate the relationship between perioperative nutritional intervention, especially preoperative intervention and surgical effect in the ERAS group. Patients receiving the perioperative nutrition regimen had a shorter hospital stay, faster recovery of intestinal function, and greater patient satisfaction compared with patients in the control group. Furthermore, immunity was enhanced and there were less postoperative complications compared with the control group. Early preoperative nutrition status was associated with a significant reduction in postoperative overall complications. According to the experimental results, the extremely low incidence of postoperative complications may be related to long-term preoperative training. This effect was more pronounced in patients who received longer periods of preoperative nutrition. In addition, their physical condition and mental outlook were better in the early postoperative period than those in the control group. This was mainly reflected in their significantly better physical condition and earlier participation in postoperative exercise recovery, and their compliance and overall satisfaction were better than those in the control group. In conclusion, we have shown that preoperative nutritional intervention played a key role in the prognosis of patients undergoing surgery.

Satisfaction evaluation was a balance between the patients’ expectations of care and the actual care provided, reflecting the changes in health status caused by the effectiveness of hospital care (26, 27). The analysis of the satisfaction test results showed that patients found value in using personalized clinical nursing measures. Furthermore, important results were through data collation: clinical compliance of the patients (such as quitting smoking and drinking, taking medication regularly, and exercising regularly) was significantly associated with patient satisfaction. Compared with the control group, no significant difference in the ERAS group was found in regard to satisfaction with clinical nursing. However, in terms of self-subjective feelings, the survey results showed that the ERAS group had more positive emotions and better expectations for both the near and distant future. This is obviously of great value to the clinical rehabilitation and follow-up treatment of patients. These predictors could be interpreted as the determinants of patient satisfaction in each group when other factors do not change greatly within the group.

The current study had some limitations. The main weakness of this study was the absence of important nutritional indices, such as calorie needs, energetic needs, protein needs etc., which we tried to compensate for with albumin and other biochemical markers. Advantages were that we used NRS score and preoperative EN before surgery, and we followed the patients nutritional support suggestions after discharge. Furthermore, while our data supported the efficacy and safety of our perioperative nutrition support program, larger multicenter studies are needed to assess its applicability in patients undergoing CEA surgery.

## Conclusion

According to our study, perioperative nutrition in ERAS program had a positive effect on postoperative rehabilitation and improved postoperative complications in CEA patients. The LOS and the cost of hospitalization were, in turn, significantly reduced. Finally, under dedicated nursing care, the mental state and subjective feelings of patients were greatly improved. Further research is needed to demonstrate the effect of clinical nutrition support in a pragmatic manner.

## Data availability statement

The original contributions presented in the study are included in the article/supplementary material, further inquiries can be directed to the corresponding authors.

## Ethics statement

The studies involving human participants were reviewed and approved by the Institutional Human Research and Ethics Committee of Tangdu Hospital. The patients/participants provided their written informed consent to participate in this study.

## Author contributions

BL and YQ conducted the study design. Y-QL, X-PQ, and L-WP completed the writing of the manuscript. Later revisions were done by BL, YQ, J-YA, X-WL, and YZ. Y-QL, X-PQ, L-WP, J-YA, X-WL, YZ, CW, XJ, LG, GL, D-LW, and D-CZ participated in the data collection, while data analysis is done by CW, XJ, LG, GL, D-LW, and D-CZ. All authors contributed to the article and approved the submitted version.

## Funding

This work was supported by the National Natural Science Foundation of China (nos. 81901188 and 81971206), the Key Research and Development Plan in Shaanxi Province of China (2016SF-041 and 2019SF-068). This research received no specific grant from any funding agency in the public, commercial, or non-profit sectors.

## Conflict of interest

The authors declare that the research was conducted in the absence of any commercial or financial relationships that could be construed as a potential conflict of interest.

## Publisher’s note

All claims expressed in this article are solely those of the authors and do not necessarily represent those of their affiliated organizations, or those of the publisher, the editors and the reviewers. Any product that may be evaluated in this article, or claim that may be made by its manufacturer, is not guaranteed or endorsed by the publisher.
